# Mental health of adolescents associated with sexual and reproductive outcomes: a systematic review

**DOI:** 10.2471/BLT.20.254144

**Published:** 2021-03-02

**Authors:** Rachel Vanderkruik, Lianne Gonsalves, Grace Kapustianyk, Tomas Allen, Lale Say

**Affiliations:** a Massachusetts General Hospital, 185 Cambridge Street, Boston, MA 02114, United States of America.; b UNDP-UNFPA-UNICEF-WHO-World Bank Special Programme of Research, Development and Research Training in Human Reproduction, Department of Sexual and Reproductive Health and Research, World Health Organization, Geneva, Switzerland.; c St Michael's Hospital, Unity Health Toronto, Toronto, Canada.; d Department of Quality, Norms and Standards, World Health Organization, Geneva, Switzerland.

## Abstract

**Objective:**

To systematically review the literature on the mental health of adolescents associated with sexual and reproductive outcomes, and compare the mental health outcomes with that of other age groups.

**Methods:**

We searched seven databases for relevant peer-reviewed articles published between 1 January 2010 and 25 April 2019. Our inclusion criteria required that the study included age-disaggregated data on adolescents, and focused and assessed mental health outcomes associated with pregnancy or sexually transmitted infections. We extracted data on the specific health event, the mental health outcome and the method of measuring this, and comparisons with other age groups.

**Findings:**

After initially screening 10 818 articles by title and abstract, we included 96 articles in our review. We observed that a wide-ranging prevalence of mental ill-health has been reported for adolescents. However, most studies of mental health during pregnancy did not identify an increased risk of depression or other mental disorders among adolescents compared with other age groups. In contrast, the majority of studies conducted during the postpartum period identified an increased risk of depression in adolescents compared with other age groups. Three studies reported on mental health outcomes following abortion, with varying results. We found no studies of the effect of sexually transmitted infections on mental health among adolescents.

**Conclusion:**

We recommend that sexual and reproductive health services should be accessible to adolescents to address their needs and help to prevent any adverse mental health outcomes.

## Résumé

**Objectif:**

Procéder à une revue systématique de la littérature consacrée aux liens entre la santé mentale des adolescents et leur santé sexuelle et reproductive, et comparer les observations relatives à la santé mentale avec celles des autres catégories d'âge.

**Méthodes:**

Nous avons examiné sept bases de données à la recherche d'articles pertinents avec comité de lecture, publiés entre le 1^er^ janvier 2010 et le 25 avril 2019. Pour répondre à nos critères d'inclusion, l'étude devait contenir des données ventilées par âge sur les adolescents, se concentrer sur la santé mentale en cas de grossesse ou d'infection sexuellement transmissible et l'évaluer. Nous avons prélevé des informations sur l'événement lui-même, sur la santé mentale et la méthode employée pour l'évaluer, mais aussi des comparaisons avec d'autres catégories d'âge.

**Résultats:**

Après avoir passé au crible le titre et le résumé de 10 818 articles, nous avons retenu 96 études pour notre revue. Nous avons constaté une grande diversité de prévalence des troubles mentaux chez les adolescents concernés. La plupart des études s'intéressant à la santé mentale pendant la grossesse n'ont toutefois pas identifié plus de risques de dépression ou d'autres troubles mentaux dans cette tranche d'âge que dans d'autres. En revanche, la majorité des études menées au cours de la période post-partum ont fait état d'un risque accru de dépression chez les adolescents par rapport à d'autres catégories. Trois études mentionnent des problèmes de santé mentale après un avortement, avec divers résultats. Nous n'avons trouvé aucune étude consacrée aux répercussions des infections sexuellement transmissibles sur la santé mentale des adolescents.

**Conclusion:**

Nous recommandons de rendre les services de santé sexuelle et reproductive accessibles aux adolescents afin de répondre à leurs besoins et de limiter l'impact négatif sur leur santé mentale.

## Resumen

**Objetivo:**

Revisar sistemáticamente las publicaciones sobre la salud mental de los adolescentes asociada a los resultados sexuales y reproductivos, y comparar los resultados de salud mental con los de otros grupos de edad.

**Métodos:**

Se realizaron búsquedas en siete bases de datos de artículos relevantes revisados por expertos y publicados entre el 1 de enero de 2010 y el 25 de abril de 2019. Nuestros criterios de inclusión requerían que el estudio incluyera datos desglosados por edad de los adolescentes, centrados y que evaluaran los resultados de salud mental asociados con el embarazo o las infecciones de transmisión sexual. Se extrajeron datos sobre el evento de salud específico, el resultado de salud mental y el método de medición de este, así como las comparaciones con otros grupos de edad.

**Resultados:**

Después de seleccionar inicialmente 10.818 artículos por título y resumen, se incluyeron 96 estudios en nuestra revisión. Observamos que se ha informado de una amplia prevalencia de mala salud mental en las adolescentes. Sin embargo, la mayoría de los estudios sobre salud mental durante el embarazo no identificaron un mayor riesgo de depresión u otros trastornos mentales entre las adolescentes en comparación con otros grupos de edad. En cambio, la mayoría de los estudios realizados durante el período posparto identificaron un mayor riesgo de depresión en las adolescentes en comparación con otros grupos de edad. Tres estudios informaron sobre los resultados de la salud mental después del aborto, con resultados variados. No se encontraron estudios sobre el efecto de las infecciones de transmisión sexual en la salud mental de las adolescentes.

**Conclusión:**

Recomendamos que los servicios de salud sexual y reproductiva sean accesibles a los adolescentes para atender sus necesidades y ayudar a prevenir cualquier resultado adverso para la salud mental.

## ملخص

الغرض إجراء مراجعة منهجية للمؤلفات المتعلقة بالصحة العقلية للمراهقين، تلك المرتبطة بالنتائج الجنسية والإنجابية، ومقارنة نتائج الصحة العقلية بمثيلاتها للفئات العمرية الأخرى.

الطريقة قمنا بالبحث في سبع قواعد بيانات للمقالات ذات الصلة، التي تمت مراجعتها بواسطة الأقران، وتم نشرها بين 1 يناير/كانون ثان 2010، و25 أبريل/نيسان 2019. تطلبت معايير الشمول بالبحث لدينا أن تتضمن الدراسة بيانات مصنفة حسب العمر عن المراهقين، وأن تكون قد ركزت على نتائج الصحة العقلية المرتبطة بالحمل أو العدوى المنقولة جنسيًا، مع تقييم هذه النتائج. قمنا باستخراج بيانات عن الحدث الصحي المحدد، ونتائج الصحة العقلية وطريقة قياس ذلك، فضلاً عن عقد المقارنات مع الفئات العمرية الأخرى.

النتائج بعد الفحص المبدئي لعدد 10818 مقالة مصنفة حسب العنوان والملخص، قمنا بتضمين 96 دراسة في مراجعتنا. لاحظنا أنه تم الإبلاغ عن انتشار واسع النطاق لاعتلال الصحة العقلية بين المراهقين. إلا أن معظم دراسات الصحة العقلية خلال فترة الحمل لم تحدد الخطر المتزايد للاكتئاب أو غيره من الاضطرابات العقلية بين المراهقين، مقارنة مع الفئات العمرية الأخرى. على النقيض من ذلك، أشارت غالبية الدراسات التي أجريت خلال فترة ما بعد الولادة إلى زيادة خطر الإصابة بالاكتئاب لدى المراهقين مقارنة مع الفئات العمرية الأخرى. أوضحت ثلاث دراسات نتائج الصحة العقلية بعد الإجهاض، مشيرة إلى نتائج متفاوتة. لم نجد دراسات حول تأثير الأمراض المنقولة جنسياً على الصحة العقلية بين المراهقين.

الاستنتاج نحن نوصي بضرورة توفير خدمات الصحة الجنسية والإنجابية للمراهقين، وذلك لتلبية احتياجاتهم، والمساعدة في الحيلولة دون وقوع أية نتائج عكسية للصحة العقلية. ****

## 摘要

**目的:**

旨在系统综述与性和生殖结果有关的青少年心理健康相关文献，并将这个阶段和其他年龄段青少年的心理健康结果进行比较。

**方法:**

我们搜索了 7 个数据库，以查找 2010 年 1 月 1 日至 2019 年 4 月 25 日期间发表的相关同行评议文章。我们的纳入标准是该研究需包含按年龄分类的青少年相关数据，并重点讨论和评估与怀孕或性传播感染有关的心理健康结果。我们提取了与特定健康事件、心理健康结果及其测量方法以及与其他年龄段的比较情况相关的数据。

**结果:**

按标题和摘要初步筛选出 10818 篇文章之后，我们将其中 96 篇研究文章纳入了综述部分。根据报道，我们发现青少年心理不健康的现象非常普遍。但是，大多数妊娠期心理健康研究发现，相对于其他年龄段，该阶段的青少年患有抑郁或其他心理疾病的风险并未增加。相反，大多数产后研究表明，相对于其他年龄段，该阶段的青少年抑郁风险有所增加。三项研究报道的流产后心理健康结果各不相同。我们未看到有关性传播感染对青少年心理健康影响的研究。

**结论:**

我们建议为青少年提供性健康和生殖健康服务，以满足其需求并帮助其预防任何不良心理健康结果。

## Резюме

**Цель:**

Провести системный обзор литературы по зависимости психического здоровья подростков от событий, имеющих последствия для их сексуального и репродуктивного здоровья, и сравнить с результатами оценки психического здоровья представителей других возрастных групп.

**Методы:**

Для поиска в целях подготовки настоящего обзора рецензированных статей, опубликованных в период между 1 января 2010 года и 25 апреля 2019 года, нами были использованы семь баз данных. Критерии включения предусматривали использование в рамках исследования данных по подросткам без разбивки по возрасту, которые бы в первую очередь касались оценки зависимости психического здоровья от беременности или инфекций, передаваемых половым путем. Нами были извлечены данные о специфических, связанных со здоровьем событиях и влиянии соответствующих событий на психическое здоровье, а также о методике оценки указанного и сравнения результатов с результатами, полученными в отношении иных возрастных групп.

**Результаты:**

После первоначального скринингового обследования по названию и тезисам 10 818 статей в обзор было включено 96 исследований. Было выявлено, что среди подростков отмечается широкая распространенность психических расстройств. Однако большинство исследований психического здоровья во время беременности не выявили повышенного риска депрессии или иных психических расстройств среди подростков в сравнении с иными возрастными группами. Напротив, большинство исследований, касающихся послеродового периода, выявили повышенный риск депрессии у подростков в сравнении с другими возрастными группами. В трех исследованиях сообщалось о состоянии психического здоровья после аборта, при этом результаты были неоднозначны. Не было обнаружено исследований о влиянии инфекций, передаваемых половым путем, на психическое здоровье подростков.

**Вывод:**

Рекомендуется обеспечить доступ подростков к услугам в области сексуального и репродуктивного здоровья для удовлетворения их потребностей и предотвращения любых неблагоприятных последствий для психического здоровья.

## Introduction

In many countries, adolescents (i.e. those aged 10–19 years) struggle to access necessary sexual and reproductive health information and services.^1^ Complications during pregnancy and childbirth are the leading cause of death globally for girls aged 15–19 years.^2^ One in four sexually active adolescents has a sexually transmitted infection, and 3 million girls aged 15–19 years undergo unsafe abortions annually.^3^

Although the effect of a sexual and reproductive health event (e.g. pregnancy or sexually transmitted infection) on an adolescent’s physical health and well-being is acknowledged,^1^ the global mental health burden that may be related to the outcomes of sexual activity is not well understood. A 2009 review conducted by the World Health Organization (WHO) identified close links between women’s sexual and reproductive health and their mental health.^4^ However, many of the participants of this review were married women of childbearing age (i.e. often not adolescents) in middle- and high-income countries. The link between the sexual and reproductive health and the mental health of men and young, single women remains largely unexplored.^4^

We therefore conducted a systematic review to examine the impact of key sexual and reproductive health events on mental health outcomes among adolescents. Specifically, we focused on events that can occur as a result of unprotected sexual activity, for example: pregnancy; the result of that pregnancy; and/or sexually transmitted infections, including human immunodeficiency virus (HIV). Our systematic review addressed two main areas: (i) the adverse mental health outcomes experienced by adolescents worldwide following key sexual and reproductive health events; and (ii) how this mental health burden among adolescents compares with that of people of other ages after experiencing the same event.

## Methods

### Search strategy

Our protocol was adapted from that of a prior systematic review of causes of maternal morbidity and mortality,^5^ and was conducted according to the Preferred Reporting Items for Systematic Reviews and Meta-Analyses (PRISMA) guidelines. We identified related publications by searching the databases PubMed®, CINAHL, Embase®, APA PsycINFO®, POPLINE, ERIC (Education Resources Information Center) and Global Index Medicus, as well the reference lists of relevant articles. We developed our search strategy for each database in collaboration with a librarian, using terms related to “mental health”, “adolescents” and “sexual and reproductive health”; we provide an example search strategy in the data repository.^6^

### Selection criteria

Our search included peer-reviewed literature published between 1 January 2010 and 25 April 2019. We selected this particular date range to capture the most recent literature, but also to build on the 2009 WHO review of the mental health aspects of women’s reproductive health.^4^

Our inclusion criteria required that the study: included age-disaggregated data on male and/or female adolescents; focused on mental health outcomes associated with either a pregnancy, the result of that pregnancy (either childbirth and the postpartum period, or an abortion) and/or horizontally transmitted sexually transmitted infections; assessed mental health outcomes that followed a sexual and reproductive health event; and was peer-reviewed. Because of the inconsistency in the literature on the exact definition of the postpartum period,^7^ we included any articles referencing the postpartum period as within one year following childbirth. In the case of a randomized controlled trial or intervention study, we also included data from the control group. 

We excluded studies that: had sample sizes less than 50; did not disaggregate adolescent-only data; did not quantify mental health outcomes; did not clarify that the sexual and reproductive health event preceded the mental health outcome of interest; used a sample group that was not representative of a general, healthy population (e.g. we excluded studies that recruited only individuals: (i) with specific pre-existing conditions such as type 1 diabetes mellitus, or a mental health condition; or (ii) exhibiting specific behaviours, such as injecting drugs); or were published in languages other than English, French, Italian, Portuguese, Spanish or Turkish.

Following the removal of duplicates, we initially screened the articles by title and abstract before screening the remaining articles in full. The two reviewers assessed and categorized articles as include, unsure or exclude, resolving discrepancies through discussion. A third reviewer, whose judgement was considered final, adjudicated unresolved disputes. 

### Data extraction

We extracted data on the general study characteristics, the specific sexual and reproductive health event and the mental health outcome of interest (e.g. relative risk among adolescents compared with other age groups). We also extracted comparison data for other age groups if it was presented in a way that assessed the statistical difference between age groups, for example, odds ratio (OR) or relative risk with 95% confidence intervals (CIs). The two reviewers who conducted the initial title and abstract screen independently extracted relevant data using an extraction form. All extracted data were double-checked and confirmed by the other reviewer, and the third reviewer resolved disagreement in the same manner as for study inclusion. Given the diversity in the study designs, measurement tools and definitions adopted in the included articles, we did not perform a meta-analysis of the findings; instead, we summarized outcomes according to type of sexual and reproductive health event.

We used a modified Joanna Briggs Institute critical appraisal checklist to assess the quality of studies reporting prevalence data.^8^ We assessed papers for quality according to eight criteria (data repository),^6^ and assigned each criterion a score of either 0 (not fulfilled), 1 (unclear whether fulfilled) or 2 (fulfilled); possible scores ranged from 0 to 16. We developed categories of quality, and considered scores of ≤ 12, of 13 or 14, and of 15 or 16 to represent studies of low, medium and high quality, respectively. We also included the stipulation that high-quality articles must score 2 points for the fifth criterion, that is, the study used objective, validated criteria to measure the mental health outcome. The same two reviewers conducted separate quality assessments for all articles, with the same third reviewer resolving differences through discussion. 

## Results

**Fig. 1:**
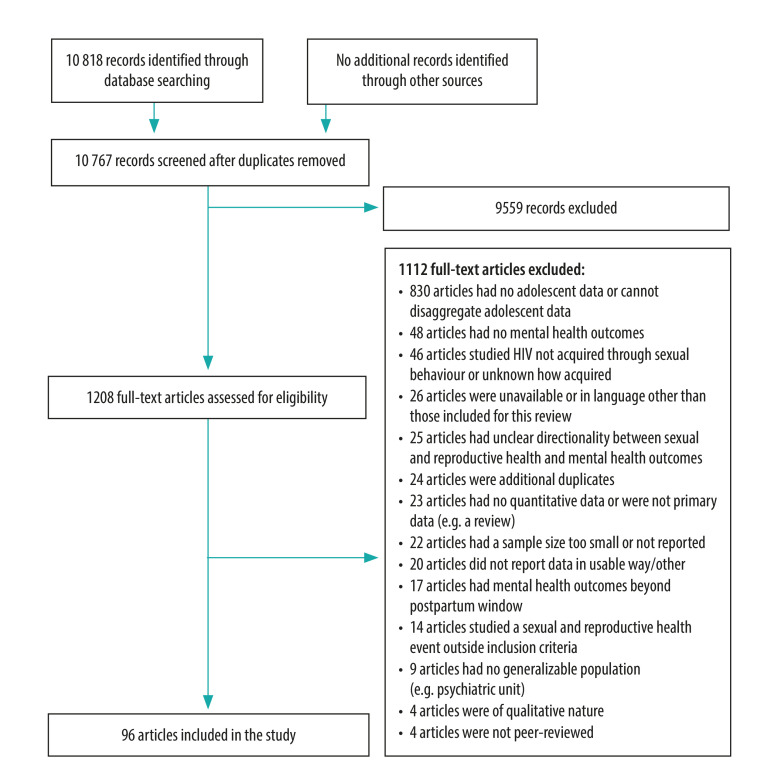
Flow chart of the selection of studies in the systematic review on mental health outcomes among adolescents following sexual and reproductive health events

We initially screened 10 818 articles by title and abstract, after removing duplicates; 9559 articles were immediately excluded. Following full-text review, we excluded another 1112 articles. We therefore included 96 articles in our review, spanning 26 different countries (Fig. 1). Forty-eight studies were conducted in high-income countries, 36 in upper-middle-income countries, 10 in lower-middle-income countries and only two in low-income countries. The United States of America was the country most represented with 38 studies, followed by Brazil with 22. Most (55 studies) were of medium quality and around one third (33 studies) were of high quality; the remainder (8 studies) were classified as being of low quality.

HIV: human immunodeficiency virus.

Around one half of the articles reported on the prevalence or mean/median of mental health conditions or symptoms during pregnancy (48 studies) and/or the postpartum period (51 studies). Three articles reported on the impact of abortion. None of our included studies reported on horizontally transmitted infections. All studies included only women in their samples with the exception of two: one study included both males and females^9^ and another included males only.^10^ We observed that a variety of tools were used to assess for mental health conditions, including symptom assessment scales or diagnostic tools or codes. Box 1 provides a summary of the tools used and the observed frequency of use among our included studies.

Box 1 **Systematic review of adolescent mental health following sexual and reproductive health events, 2020: frequency of mental health assessment tools used in 96 included studies**

**Symptom scales for depression**

Edinburgh Postnatal Depression Scale (EPDS) or its short form (EPDS-6): 40 studies

Center for Epidemiologic Studies Depression scale (CES-D, CES-D Children; also known as CES-D20 or CES-D30, depending on number of items): 18 studies

Original Beck Depression Inventory (BDI) or latest version updated to incorporate cognitive, affective, somatic and vegetative symptoms of depression (BDI II): 4 studies

Other (adapted/modified from other tools): 3 studies

9- or 2-item Patient Health Questionnaire (PHQ-9 or PHQ-2): 2 studies

Children’s Depression Rating Scale, Revised (CDRS-R): 2 studies

Delusions-Symptoms-States Inventory: State of Anxiety and Depression (DSSI/SAD): 1 study

**Symptom scales for anxiety or stress**

State–Trait Anxiety Inventory (STAI): 3 studies

Revised Children’s Manifest Anxiety Scale (RCMAS): 1 study

Beck Anxiety Inventory (BAI): 1 study

PTSD (post-traumatic stress disorder) Checklist – Civilian Version (PCL-C): 1 study

14-item Perceived Stress Scale (PSS-14): 1 study

**Symptom scales for common mental conditions**

20-item Self-Reporting Questionnaire (SRQ-20): 3 studies

General Health Questionnaire (GHQ): 1 study

**Diagnostic tools or codes**

International Statistical Classification of Diseases and Related Health Problems (ICD-9 or 10) codes: 6 studies

Mini-International Neuropsychiatric Interview (MINI): 6 studies

Structured clinical interview for DSM-IV (Diagnostic and Statistical Manual of Mental Disorders, fourth edition) childhood disorders (Kid-SCID): 3 studies

Pregnancy Risk Assessment Monitoring System (PRAMS): 2 studies

Composite International Diagnostic Interview (CIDI): 2 studies

Diagnostic and Statistical Manual of Mental Disorders, fourth edition (DSM-IV): 2 studies

Schedule for Affective Disorders and Schizophrenia for School-age children, present and lifetime version (KSADS-PL) clinical interview: 1 study

Clinical Interview Schedule, Revised (CIS-R): 1 study

Primary Care Evaluation of Mental Disorders (PRIME-MD): 1 study

Zung scale: 1 study

### Pregnancy

Of the 48 studies focusing on the prevalence or mean/median of mental health conditions or symptoms during pregnancy (Table 1 available at: http://www.who.int/bulletin/volumes/99/5/20-254144), the majority (37 studies) reported on depression. Most of these (28 studies) reported the prevalence of depression or depressive symptoms (having at least mild symptoms during pregnancy) ranging from 2.0%^39^ to 89.1%.^41^ Nine studies reported mean or median depression scores.

**Table 1:** Systematic review of adolescent mental health following sexual and reproductive health events, 2020: studies of pregnancy

Author, year	Country	Study population	Age (years)	No. adolescents	Sexual and reproductive health event time frame	Assessment tool	Outcome reported	Outcome estimate	Quality rating
**Depression**									
Fagan & Lee 2010^a^^11^	USA	Pregnant adolescents, with expectant father age < 24 years	13–19^b^	100	5–9 months pregnant	CES-D (higher score indicates more depressive symptoms)	Mean (SD)	34.96 (7.99)	High
Jansen et al. 2010^a^^12^	Brazil	Pregnant women attending any public health-care unit in an urban area	12–19	287	Not specified	EPDS (≥ 13)	Prevalence	18.8%	High
Pereira et al. 2010^13^	Brazil	Adolescents attending prenatal care at a health centre	10–19^b^	120	Trimester 3	CIDI	Prevalence	14.20%	High
Silva et al. 2010^14^	Brazil	Pregnant women attending public sector services	12–18	232	Not specified	EPDS (≥ 13)	Prevalence	18.50%	Medium
Nasreen et al. 2011^a^^15^	Bangladesh	Pregnant women, rural	< 20	157	Trimester 3	EPDS (≥ 10)	Prevalence	14.00%	High
Carvajal et al. 2012^16^	USA	Adolescents attending clinics, primarily low-income and minorities	< 19^b^	164	Trimester 3	CES-D (≥ 24)	Prevalence	17%	Medium
Chalem et al. 2012^a^^17^	Brazil	Adolescents attending prenatal care at hospital	12–19^b^	457	Not specified	CIDI 2.1	Prevalence	13.50%	Medium
East et al. 2012^a^^18^	USA	First-time pregnant adolescents, unmarried, Mexican-American	15–19^b^	100	Trimester 3	CES-D	Mean (SD)	2.60 (1.14)	Medium
Lanzi et al. 2012^a^^19^	USA	First-time pregnant adolescents, 76% African–American	15–19^b^	270	Trimesters 2 and 3	BDI	Mean (SD)	13.19^c^	High
Lara et al. 2012^a^^20^	Mexico	Pregnant and parenting Mexican adolescents	13–19^b^	8 049 088^d^	Trimesters 1, 2 and 3	CES-D (depressive symptoms: 16–23; high symptomatology: ≥ 24)	Prevalence	CES-D (16–23): Trimester 1: 17.3%; trimester 2: 32.5%; trimester 3: 8.2% CES-D (≥ 24): Trimester 1: 11.4%; trimester 2: 5.4%; trimester 3: 10.8%	High
Pinheiro et al. 2012^a^^21^	Brazil	Adolescents attending prenatal care in public health system	13–19^b^	828	Not specified (mean gestational age: 23.1 weeks)	MINI (Portuguese version)	Prevalence	17.80%	Medium
Tzilos et al. 2012^22^	USA	Adolescents attending prenatal clinic in an urban area	13–18^b^	116	Not specified (mean gestational age: 20 weeks)	CDRS-R (> 40)	Mean (SD)	53.5 (6.5)	High
Williams et al. 2012^e^^10^	USA	First-time adolescent fathers, African–American	14–19^b^	59	Partner in trimester 3	CES-D (high level of symptoms ≥ 16; clinical depression range ≥ 23)	Mean (SD)	11.78 (8.38)	Low
Coelho et al. 2013^23^	Brazil	Adolescents attending prenatal care through public health system	13–19^b^	828	Not specified (mean gestational age: 23.1 weeks)	MINI (Portuguese version)	Prevalence	17.80%	Medium
Nunes & Phipps 2013^a^^24^	USA	Women who had recently given birth, Rhode Island, population-representative survey	15–19	676	Not specified	Modified PHQ-2	Prevalence	8.44%^c^	Low
Alvarado-Esquivel et al. 2014^25^	Mexico	Adolescents attending prenatal care in a public hospital	13–17^b^	120	Trimesters 1, 2 and 3	DSM-IV	Prevalence	Minor: 20.83%; major: 1.67%	Medium
Ertel et al. 2014^26^	USA	Women of Puerto Rican or Dominican Republic heritage	16–19	303	Trimesters 1 and 2	EPDS (minor: ≥ 13; major: ≥ 15)	Prevalence	Minor: 22.44%; major: 14.19%	High
Pires et al. 2014^27^	Portugal	Pregnant adolescents	12–19^b^	395	Not specified (mean: 24 weeks)	EPDS (> 9)	Mean (SD)	7.00 (4.90)	Medium
Weobong et al. 2014^a^^28^	Ghana	Pregnant women identified during December 2007–June 2009	15–19	2 360	Not specified	PHQ-9 (major: ≥ 5; minor: 2–4)	Prevalence	10.1% (combined minor and major depression)	High
Alvarado-Esquivel et al. 2015^29^	Mexico	Women who attended prenatal care at a public hospital	13–17^b^	181	Trimesters 1, 2 and 3	EPDS (≥ 8); DSM-IV	Prevalence	20.44%	High
Bonilla-Sepúlveda 2015^a^^30^	Colombia	Women receiving high-risk obstetric service from a tertiary public hospital serving low-income populations	10–19^b^	124	Not specified	Zung scale (none: < 50; mild: 51–59; moderate: 60–69; severe: > 69)	Prevalence	Mild: 17.6%; moderate: 10.4%; severe: 4.8%	Medium
Buzi et al. 2015^31^	USA	Adolescents attending community-based teen health clinic providing free/low-cost care or referred by a school or community group	15–18^b^	249	Not specified	CES-D (≥ 16)	Prevalence	46.10%	High
Uthaipaisanwong et al. 2015^32^	Thailand	Pregnant adolescents attending hospital services	13–19^b^	200	Trimesters 1, 2 and 3	EPDS (≥ 11)	Prevalence	46%	Medium
Zeiders et al. 2015^a^^33^	USA	Unmarried adolescent mothers in urban area, Mexican origin	15–18^b^	204	Trimester 3	CES-D (higher score indicates more depressive symptoms)	Mean (SD)	17.98 (10.17)	Medium
Målqvist et al. 2016^a^^34^	Eswatini	Pregnant women in the community	14–19	179	Trimester 3	EPDS (≥ 13)	Prevalence	24.60%	High
Samankasikorn et al. 2016^a^^35^	USA	Pregnant adolescents recruited from three locations (two urban, one rural)	Teenagers^b^	66	Not specified (mean gestational age: 18 weeks)	EPDS (12, 13)	Mean (SD)	6.76 (4.32)	Medium
Coll et al. 2017^a^^36^	Brazil	Women with estimated delivery dates during December 2014–May 2016	< 20	603	Trimester 2	EPDS (≥ 13)	Prevalence	21.00%	High
Faisal-Cury et al. 2017^a^^37^	Brazil	Pregnant women recruited from 10 public primary care clinics in São Paulo	16–19	147	Trimesters 2 and 3	SRQ-20 (> 7)	Prevalence	19.7%	Medium
Szegda et al. 2017^38^	USA	Latina attendees of prenatal care at tertiary care centre in Massachusetts, 2006–2011	16–19	270	Not specified (mean gestational age: 25.7 weeks)	EPDS (probable minor depression: ≥ 13; probable major depression: ≥ 15)	Prevalence	Minor: 28.8%; major: 26.9%	Medium
Abdelaal et al. 2018^39^	USA	National database of primary and tertiary care hospitals, pregnant adolescents seeking care	13–19^b^	1 023 586^d^	Trimester 3 (point of admission)	ICD-9 codes	Prevalence	2.01% (in 2012)	High
Bernard et al. 2018^40^	Jamaica	Nationwide survey of women who gave birth during July–September 2011	< 20	1 853 (721 with available EPDS scores)	Trimesters 1, 2 and 3	EPDS (≥ 13)	Prevalence	Low likelihood (≤ 9): 65.5%; moderate likelihood (10–12): 13.5%; high likelihood (≥ 13): 21%	Medium
Kimbui et al. 2018^41^	Kenya	Pregnant adolescents, peri-urban	14–18^b^	212	Throughout pregnancy	EPDS Kisawa Hili translation (≥ 8) BDI II	Prevalence	Mild: 10.8%; moderate: 26.4%; severe: 51.9%	Medium
Osok et al. 2018^42^	Kenya	Attendees of maternal and child health clinic, likely low- to middle-income and from an informal settlement	15–18^b^	176	Not specified	EPDS (≥ 13) PHQ-9 (≥ 15)	Prevalence	EPDS: 58% Subsequent PHQ-9 screen: 33%	Medium
Phoosuwan et al. 2018^a^^43^	Thailand	Women attending prenatal services in hospital	< 20	79	Trimester 3	EPDS (≥ 10)	Prevalence	59.50%	High
Salehi-Pourmehr et al. 2018^a^^44^	Iran (Islamic Republic of)	Pregnant women of healthy weight and with BMI ≥ 35	15–19	64	All trimesters	EPDS (> 12)	Median (min, max)	Trimester 1: 7 (0, 22); trimester 2: 6 (0, 17); trimester 3: 7 (0, 20)	Low
Surkan et al. 2018^a^^45^	Bangladesh	Married women of a reproductive age	< 20	5 742	Trimester 3	Tool adapted from PHQ-9 and CES-D	Prevalence	7%	Medium
Duko et al. 2019^a^^46^	Ethiopia	Women attending prenatal care clinics	15–19	108	Not specified	EPDS (> 13)	Prevalence	10.20%	High
**Anxiety**									
Chalem et al. 2012^a^^17^	Brazil	Adolescents attending prenatal care at hospital	12–19^b^	457	Not specified	CIDI 2.0	Prevalence	Post-traumatic stress disorder: 10.5%; anxiety: 4.6%	Medium
East et al. 2012^a^^18^	USA	First-time pregnant adolescents, unmarried, Mexican–American	15–19^b^	100	Trimester 3	RCMAS	Mean (SD)	2.80 (1.10)	Medium
Pinheiro et al. 2012^a^^21^	Brazil	Adolescents attending prenatal care in public health system	13–19^b^	828	Not specified (mean gestational age: 23.1 weeks)	MINI (Portuguese version)	Prevalence	Generalized anxiety disorder: 8.7%; obsessive–compulsive disorder: 3.5%; panic disorder: 2.2%; post-traumatic stress disorder: 2.5%; social anxiety disorder: 5.1%; any anxiety disorder: 13.6%; comorbid depression and anxiety: 9.1%	Medium
Coelho et al. 2014^a^^47^	Brazil	Adolescents attending prenatal care through public health system	13–19^b^	828	Not specified (mean gestational age: 23.1 weeks)	MINI (Portuguese version)	Prevalence	Generalized anxiety disorder: 8.7%; obsessive–compulsive disorder: 3.5%; panic disorder: 2.2%; post-traumatic stress disorder: 2.5%; social anxiety disorder: 5.1%	Medium
Fonseca-Machado et al. 2015^48^	Brazil	Women in trimester 3 attending prenatal care at a clinic during May 2012–May 2013	15–19	78	Trimester 3	PCL-C, STAI	Prevalence, mean	Post-traumatic stress disorder: 19.2%; mean trait score: 41.6; mean state score: 43.6	High
Barcelona de Mendoza et al. 2016^49^	USA	Pregnant women of Puerto Rican or Dominican Republic heritage, attending public obstetrics clinic	16–19	441	Trimesters 1, 2 and 3	STAI	Mean (SD)	Early pregnancy: 39.8 (9.8); mid-pregnancy: 33.5 (10.8); late pregnancy: 32.4 (10.0)	Medium
Peter et al. 2017^f^^50^	Brazil	Adolescents attending prenatal care in public health system	10–19^b^	871	Not specified	MINI (Portuguese version)	Prevalence	Any anxiety disorder: 13.6%; panic disorder: 2.1%; social phobia: 2.8%; post-traumatic stress disorder: 2.4%; obsessive–compulsive disorder: 3.1%; generalized anxiety disorder: 8.7%	Medium
Matos et al. 2018^51^	Brazil	Adolescents attending prenatal care in public health system in an urban area	≤ 16, 17–19^b^	870 (≤ 16 years: 240; 17–19 years: 630)	Pregnancy	MINI	Prevalence	16.19%^c^	Medium
Salehi-Pourmehr et al. 2018^a^^44^	Iran (Islamic Republic of)	Pregnant women of healthy weight and with BMI ≥ 35	15–19	64	Trimesters 1, 2 and 3	BAI	Median (min, max)	Trimester 1: 3 (0, 29); trimester 2: 3.5 (0, 33); trimester 3: 4 (0, 34)	Low
**General mental disorders/psychiatric distress**									
Faisal-Cury et al. 2010^a^^52^	Brazil	Women attending prenatal care in the public health system in São Paulo	16–19	166	Trimester 2	CIS-R (> 12)	Prevalence	30.10%	High
Silva et al. 2010^a^^53^	Brazil	Pregnant women attending public sector services	12–18^b^	232	Trimesters 2 and 3	SRQ-20 (≥ 7)	Prevalence	40.50%	Medium
Almeida et al. 2012^a^^54^	Brazil	Women receiving primary health care in Southern Brazil	< 20	181	Trimesters 2 and 3	PRIME-MD	Prevalence	37%	Medium
Chalem et al. 2012^a^^17^	Brazil	Adolescents attending prenatal care at hospital	12–19^b^	457	Not specified	CIDI 2.0	Prevalence	22.50%	Medium
Pinheiro et al. 2012^a^^21^	Brazil	Adolescents attending prenatal care in public health system	13–19^b^	828	Not specified (mean gestational age: 23.1 weeks)	MINI	Prevalence	Any psychiatric disorder: 23.9%; mania: 3.7%; hypomania: 2.8%	Medium
Suzuki 2019^a^^55^	Japan	Primiparous women aged 13–17 and 28–30 years who delivered at one maternity hospital during 2002–2016	13–17	325	Trimesters 2 and 3	Psychiatrist diagnosis	Incidence	4.92%	Medium
**Suicidality**									
Huang et al. 2012^a^^56^	Brazil	Women attending prenatal care in primary health units in São Paulo	16–19	168	During weeks 20–30 of gestation	SRQ-20	Prevalence	8.9%	Medium
Pinheiro et al. 2012^a,f^^21^	Brazil	Adolescents attending prenatal care in public health system	13–19^b^	828	Not specified (mean gestational age: 23.1 weeks)	MINI	Prevalence	Suicide behaviour: 13.3%; thoughts of self-harm: 4.2%; high risk: 3.4%; moderate risk: 1.3%; low risk: 8.6%	Medium
Coelho et al. 2014^a,f^^47^	Brazil	Adolescents attending prenatal care through public health system	13–19^b^	828	Not specified	MINI (Portuguese version)	Prevalence	Suicide behaviour: 13.3%; thoughts of self-harm: 4.2%; high risk: 3.4%; moderate risk: 1.3%; low risk: 8.6%	Medium
Zhong et al. 2018^57^	USA	Women aged 12–55 years, who delivered in hospital	12–18	1 242 318^d^	Not specified	ICD-9-CM codes	Prevalence	0.012% (147/1 242 318)^c^	Medium

BAI: Beck Anxiety Inventory; BDI: Beck Depression Inventory; BMI: body mass index; CDRS-R: Children’s Depression Rating Scale, Revised; CES-D: Center for Epidemiologic Studies Depression scale; CIDI: Composite International Diagnostic Interview; CIS-R: Clinical Interview Schedule, Revised; DSM-IV: Diagnostic and Statistical Manual of Mental Disorders, fourth edition; EPDS: Edinburgh Postnatal Depression Scale; ICD: International Statistical Classification of Diseases and Related Health Problems; ICD-9-CM: ICD, Ninth Revision, Clinical Modification; MINI: Mini-International Neuropsychiatric Interview; PCL-C: PTSD Checklist, Civilian Version; PHQ-2/-9: 2-/9-item Patient Health Questionnaire; PRIME-MD: Primary Care Evaluation of Mental Disorders; RCMAS: Revised Children’s Manifest Anxiety Scale; SD: standard deviation; SRQ-20: 20-item Self-Reporting Questionnaire; STAI: State–Trait Anxiety Inventory.

^a^ This publication is also included in other table(s).

^b^ Study population was adolescents only.

^c^ Our calculations.

^d^ Weighted sample.

^e^ Sample included males.

^f^ Pinheiro et al. 2012,^21^ Coelho et al. 2014^47^ and Peter et al. 2017^50^ used the same study populations.

Of the nine studies (four of which also reported on depression) reporting on some type of anxiety disorder or symptoms during pregnancy, six studies provided prevalence, and the prevalence of at least mild symptoms or an anxiety disorder (including post-traumatic stress disorder) ranged from 13.6%^21^ to 19.2%.^48^ Four studies reported on mean or median anxiety scores. Six studies (three of which also reported on depression and/or anxiety) reported on broad mental disorders or common mental disorders or stress during pregnancy, with prevalence ranging from 22.5%^17^ to 40.5%.^53^ One study reported an incidence rate of 4.9%.^55^ Four studies (one of which also reported on depression, anxiety and general mental disorders) reported on suicidal ideation or behaviour. The prevalence of any suicidal ideation (including thoughts of self-harm or wishes to be dead) ranged from 4.2%^21^^,^^47^ to 8.9%,^56^ while the prevalence of any suicidal behaviour ranged from < 0.1% (147/1 242 318)^57^ to 13.3%.^21^^,^^47^

Eleven studies provided data regarding depression among adolescents compared with other age groups during pregnancy (Table 2). Of these, only three studies identified an increased risk of depression among pregnant adolescents when compared with older age groups; eight studies reported no increased risk. Five studies reported comparison data regarding general mental disorders or psychological distress among adolescents compared with other age groups during pregnancy. Again, the majority of these studies (four studies) did not identify an increased risk of general mental health problems during pregnancy among adolescents compared with other age groups. One study reported on adjusted OR for suicidal ideation during pregnancy, and found adolescents to be at greatest risk compared with other age groups.^56^

**Table 2:** Systematic review of adolescent mental health following sexual and reproductive health events, 2020: studies comparing adolescents with other age groups during pregnancy

Author, year	Country	Assessment tool	Sexual and reproductive health event time frame	Outcome reported	Adolescent age (years)	No. adolescents	Adolescent outcome estimate	No. in comparison group	Comparison group outcome estimate	Quality rating
**Depression**										
Jansen et al. 2010^a^^12^	Brazil	EPDS (≥ 13)	Not specified	Adjusted PR (95% CI)	12–19	287	1.0 (–)	974	20–34 years: 1.42 (1.08–1.87); 35–49 years:1.73 (1.18–2.54)	High
Shen et al. 2010^58^	USA	ICD-9 codes	Trimester 3	Crude OR (95% CI)	15–19	90 393^b^	0.81 (0.75–0.89)	787 206^b^	20–24 years: 0.90 (0.85–0.96); 25–29 years: 1.00 (–); 30–34 years: 1.08 (1.01–1.14); 35–39 years: 1.19 (1.11–1.28); ≥ 40 years: 1.30 (1.15–1.47)	High
Silva et al. 2010^a^^53^	Brazil	EPDS (≥ 13)	Not specified	Crude PR (95% CI)	12–18	232	1.0 (–)	1032	19–34 years: 1.14 (0.85–1.54); 35–45 years: 1.44 (0.96–2.15)	Medium
Nasreen et al. 2011^a^^15^	Bangladesh	EPDS (≥ 10)	Trimester 3	Adjusted OR (95% CI)	< 20	157	1.0 (–)	563	20–34 years: 1.48 (0.71–3.06); ≥ 35 years: 3.00 (1.12–8.01)	High
Weobong et al. 2014^a^^28^	Ghana	PHQ-9 (minor: 2–4; major: > 5)	Trimesters 1, 2 and 3	Adjusted RR (95% CI)	15–19	2 360	1.01 (0.87–1.16)	18 560	20–29 years: 1.0 (–); ≥ 30 years: 1.21 (1.11–1.33)	High
Bonilla-Sepúlveda 2015^a^^30^	Colombia	Zung scale (none: < 50; mild: 51–59; moderate: 60–69; severe: > 69)	Not specified	Crude OR (95% CI)	10–19^c^	124	2.42 (1.28–4.6)	125 not pregnant (same ages)	1.0 (–)	Medium
Målqvist et al. 2016^a^^34^	Eswatini	EPDS (≥ 13)	Trimester 3	Crude OR (95% CI)	14–19	179	1.13 (0.78–1.65)	841	≥ 20 years: 1.0 (–)	High
Coll et al. 2017^a^^36^	Brazil	EPDS (≥ 13)	Trimester 2	Adjusted PR (95% CI)	< 20	603	1.0 (–)	3527	20–34 years: 1.21 (1.01–1.45); ≥ 35 years: 1.36 (1.06–1.73)	High
Phoosuwan et al. 2018^a^^43^	Thailand	EPDS (≥ 10)	Trimester 3	Adjusted OR (95% CI)	< 20	79	2.58 (1.14–5.84)	368	20–29 years: 1.30 (0.74–2.26); 30–39 years: 1.0 (–); ≥ 40 years: 1.30 (0.34–4.98)	High
Surkan et al. 2018^a^^45^	Bangladesh	Adapted from PHQ-9 and CES-D	Not specified	Adjusted RR (95% CI)	< 20	5 742	1.0 (–)	7675	20–29 years: 0.94 (0.80–1.11); ≥ 30 years: 1.38 (1.12–1.70)	Medium
Duko et al. 2019^a^^46^	Ethiopia	EPDS (> 13)	Not specified	Adjusted OR (95% CI)	15–19	108	1.0 (–)	209	20–30 years: 5.85 (3.70–10.14); > 30 years: 3.91 (0.83–8.44)	High
**General mental disorders/psychological distress**										
Faisal-Cury et al. 2010^a^^52^	Brazil	CIS-R (> 12)	Trimester 2	Crude OR (95% CI)	16–19	166	1.0 (–)	662	20–29 years: 1.27 (0.86–1.86); 30–44 years: 1.14 (0.73–1.78)	High
Witt et al. 2010^59^	USA	ICD-9 codes	Not specified	Adjusted OR (95% CI)	14–19	249	0.73 (0.32–1.65)	2484	20–24 years: 0.70 (0.34–1.43); 25–29 years: 1.41 (0.74–2.68); 30–34 years: 1.0 (–); ≥ 35 years: 1.69 (0.76–3.75)	Medium
Almeida et al. 2012^a^^54^	Brazil	PRIME-MD	Trimesters 2 and 3	Crude PR (95% CI)	< 20	181	0.92 (0.71–1.20)	531	20–29 years: 1.12 (0.90–1.39); ≥ 30 years: 1.0 (–)	Medium
Silveira et al. 2013^60^	USA	PSS-14 (> 30)	Trimesters 1, 2 and 3	Adjusted OR (95% CI)	< 19	211	0.6 (0.4–0.9)	768	19–23 years: 1.0 (–); 24–29 years: 1.2 (0.8–1.8); ≥ 30 years: 0.7 (0.4–1.3)	Medium
Suzuki 2019^a^^55^	Japan	Psychiatrist’s diagnosis	Trimesters 2 and 3	Prevalence (χ^2^)	≤ 18	325	4.90%	2029	28–30 years: 2.20% (*P* < 0.01)	Medium
**Suicidality**										
Huang et al. 2012^a^^56^	Brazil	SRQ-20	During weeks 20–30 of gestation	Adjusted OR (95% CI)	16–19	168	1.0 (–)	663	20–29 years: 0.62 (0.3–1.27); 30–44 years: 0.39 (0.15–1.07)	Medium

CES-D: Center for Epidemiologic Studies Depression scale; CI: confidence interval; CIS-R: Clinical Interview Schedule, Revised; EPDS: Edinburgh Postnatal Depression Scale; ICD: International Statistical Classification of Diseases and Related Health Problems; OR: odds ratio; PHQ-9: 9-item Patient Health Questionnaire; PR: prevalence ratio; PRIME-MD: Primary Care Evaluation of Mental Disorders; PSS-14: 14-item Perceived Stress Scale; RR: relative risk; SRQ-20: 20-item Self-Reporting Questionnaire.

^a^ This publication is also included in other table(s).

^b^ Our calculations.

^c^ Study population was adolescents only.

### Postpartum

In the 49 studies that reported on the prevalence or mean/median of mental health conditions during the postpartum period (Table 3; 47 studies; available at: http://www.who.int/bulletin/volumes/99/5/20-54144) or during both pregnancy and the postpartum period (Table 4; 2 studies), we noted that the postpartum period was defined as being as short as 72 hours to as long as 1 year after delivery. The majority of these studies (46/49) reported on depression, most (38/46) reporting prevalence of depression or depressive symptoms (i.e. having at least mild symptoms at some time during the postpartum period) from 2.5%^88^ to 57.0%.^70^ Two studies reported on incidence, which was found to be 25.0% (95% CI: 13.2–36.8%) in one study,^74^ and 8.3% at 6 weeks, 5.2% at 3 months and 6.2% at 6 months postpartum in the other.^77^ Seven studies reported mean or median symptom scores. One study (Table 4) reported on the prevalence of depression during both pregnancy and the postpartum period.^97^

**Table 3:** Systematic review of adolescent mental health following sexual and reproductive health events, 2020: studies of postpartum period

Author, year	Country	Study population	Age (years)	No. adolescents	Sexual and reproductive health event timeframe	Assessment tool	Outcome reported	Outcome estimate	Quality rating
**Depression**									
Amr & Hussein Balaha 2010^a^^61^	Saudi Arabia	Primigravid adolescents attending postnatal care within 2 months of delivery	15–19^b^	190	Within 2 months	MINI 5.0	Prevalence	Depressive disorders: 6.3% (major: 2.6%; dysthymia: 3.7%)	High
Anderson 2010^62^	USA	Adolescents attending urban, public hospital in the south-west, majority Hispanic	13–19^b^	141	Within 72 hours postpartum; 3 months	EPDS (mild: 10–12; moderate/severe: ≥ 13); CES-D (> 16)	Prevalence	EPDS (72 hours: 32.6%; 3 months: 24%) CES-D (72 hours: 30.7%)	Low
Anderson & Logan 2010^63^	USA	Adolescents self-identifying as Hispanic	13–19^b^	85	Within 72 hours	EPDS (mild: 10–12; moderate/severe: ≥ 13); CES-D (> 17)	Prevalence	EPDS (mild: 9.2%; moderate/severe: 23.3%) CES-D (symptoms: 24.6%)	Medium
Bodur et al. 2010^64^	Turkey	Adolescents attending prenatal care	15–18^b^	135	4 weeks	EPDS (> 13)	Prevalence	41.00%	Medium
Fagan & Lee 2010^a^^11^	USA	Pregnant adolescents, with expectant father aged < 24 years	13–19^b^	100	3 months	CES-D	Mean (SD)	34.79 (9.92)	High
Logsdon & Myers 2010^65^	USA	Adolescents attending a teen parent programme	13–18^b^	59	4–6 weeks	CES-D20 (> 16); CES-D30 (> 24); EPDS (> 12); KSADS-PL clinical interview	Prevalence	CES-D20: 32.2%; CES-D30: 30.5%; EPDS: 12.5%; KSADS-PL: 16.9%	High
Ramos-Marcuse et al. 2010^66^	USA	First-time adolescent mothers attending urban hospital, African–American	13–18^b^	177	Within 3 weeks; 6 months	BDI (> 9)	Prevalence	Within 3 weeks: 49%; 6 months: 37%	High
Warren et al. 2010^a^^67^	USA	Nationally representative survey of United States adolescents in secondary school in 1994–1995	13–18^b^	69	Immediately post-delivery; 1 year	CES-D (> 22)	Prevalence	Immediately post-delivery: 24.3%; 1 year: 18.2%	High
de Castro et al. 2011^68^	Mexico	Women with babies attending routine care at public paediatric units	14–19	81	Within 9 months	EPDS (> 13)	Prevalence	16.05%	Medium
Ahmed et al. 2012^69^	Iraq	Puerperal women aged 14–48 years	14–19	75	6–8 weeks	EPDS (> 10)	Prevalence	18.70%	Medium
Almeida et al. 2012^a,c^^9^	Brazil	Fathers and mothers of live births during March–December 2008, in the public health system	13–19	63	Not specified (within 1 year)	EPDS (> 13)	Prevalence	12.70%	Medium
Brown et al. 2012^70^	USA	Adolescents attending an urban hospital	< 19^b^	120	Within 1 year	CES-D (> 16)	Prevalence	57.00%	Medium
Chittleborough et al. 2012^a^^71^	United Kingdom	Women in Avon with an expected delivery date during April 1991–December 1992	< 20	655	8 weeks	EPDS (> 12)	Prevalence	6.50%	Medium
East et al. 2012^a^^18^	USA	First-time pregnant adolescents, unmarried, Mexican–American	15–19^b^	100	6 weeks; 1 year	CES-D	Mean (SD)	6 weeks: 2.32 (1.05); 1 year: 2.59 (1.17)	Medium
Lanzi et al. 2012^a^^19^	USA	First-time pregnant adolescents, 76% African–American	15–19^b^	270	6 months	BDI	Mean (SD)	No PREP risk: 8.34 (7.39); PREP risk: 11.34 (8.66)	High
Lara et al. 2012^a^^20^	Mexico	Pregnant and parenting Mexican adolescents	13–19^b^	8 049 088^d^	0–6 months; 7–12 months	CES-D (depressive symptoms: 16–23; high symptomatology: ≥ 24)	Prevalence	CES-D (16–23): 0–6 months, 2.3%; 7–12 months, 13.6% CES-D (≥ 24): 0–6 months, 4.4%; 7–12 months, 3.0%	High
Silva et al. 2012^a^^72^	Brazil	Women attending public prenatal care sector services in Pelotas	13–19	215	Within 30–60 days	EPDS (> 13)	Prevalence	19.50%	Medium
Surkan et al. 2012^73^	USA	Nationwide survey of children born in 2001 and followed prospectively through 2007	15–19	500	9 months	CES-D (mild: 5–9; moderate/severe: > 10)	Prevalence	Mild: 29.0%; moderate/severe: 26.8%	Medium
Nunes & Phipps 2013^a^^24^	USA	Women with recent deliveries in Rhode Island, population-representative survey	15–19	676	Within 10 months	Modified PHQ-2	Prevalence	Mild paranoid personality disorder: 30.37%; moderate/severe paranoid personality disorder: 2.11%	Low
Phipps et al. 2013^74^	USA	Adolescents attending an urban, prenatal clinic	14–18^b^	52	3 months; 6 months	Kid-SCID	Incidence (95% CI)	25.0 (13.2–36.8)%	Medium
Molero et al. 2014^75^	Venezuela (Bolivarian Republic of)	Late postpartum women attending an urban hospital	14–18	50	Not specified (mean: 20 days)	EPDS (without risk: < 10; limited risk: 10–12; likely depression: ≥ 13)	Prevalence, mean (SD)	Without risk: 96%; limited risk: 0%; probable/likely depression: 4% Average EPDS: 5.88 (1.96) EPDS domains: dysphoria, 12.2%; anxiety, 25.5%; feelings of guilt, 4.1%; difficulty concentrating, 1%; suicidal thoughts: 0%	Low
Venkatesh et al. 2014^76^	USA	Adolescents with pregnancy < 25 weeks attending an urban prenatal clinic	13–18^b^	106	Within 6 months	Kid-SCID (major depressive disorder); CDRS-R (sub-threshold depression: ≥ 29)	Prevalence	Major depressive disorder: 19% Sub-threshold depression: 30%	Medium
Venkatesh et al. 2014^77^	USA	Adolescents with pregnancy < 25 weeks attending an urban prenatal clinic	13–18^b^	106	6 weeks; 3 months; 6 months	Kid-SCID	Incidence, prevalence	6 weeks: incidence, 8.3%; 3 months: prevalence, 11.5%; incidence, 5.2%; 6 months: prevalence, 12.4%; incidence: 6.2%	Medium
Brito et al. 2015^78^	Brazil	Participants of the Brazilian Family Health Strategy in one district in Recife	18–19	146	Not specified	EPDS (> 12)	Prevalence	22.60%	Low
de Castro et al. 2015^a^^79^	Mexico	Women attending postnatal care in a public hospital in Mexico City	14–19	120	Within 1 year	EPDS (> 12)	Prevalence	9.20% (11/120)^e^	High
Kingsbury et al. 2015^a^^80^	Australia	Pregnant women attending one maternity hospital in Brisbane during 1981–1983	14–19	345	6 months	DSSI/SAD-7	Prevalence	29.60%	Medium
Lewin et al. 2015^81^	USA	Adolescent mother–child dyads attending primary health-care clinics, primarily urban, low-income African–Americans	13–19^b^	119	Within 6 months	CES-D (> 16)	Prevalence	28.60%	High
Milanés et al. 2015^82^	Colombia	Adolescents who gave birth at primary care centres in Cartagena in 2011	10–19^b^	460	Up to 7 days post-delivery	EPDS (no cut-off presented)	Prevalence	49.60%	High
Weobong et al. 2015^83^	Ghana	Pregnant women identified during December 2007–June 2009	15–19	1 511	Not specified	PHQ-9 (> 10)	Prevalence	3.40%	Medium
Zeiders et al. 2015^a^^33^	USA	Unmarried adolescent mothers in urban area, Mexican origin	15–18^b^	204	7–10 months	CES-D	Mean (SD)	17.29 (11.11)	Low
Anderson & Rahn 2016^84^	USA	Adolescents attending urban, public hospital, majority Hispanic	13–19^b^	260	Not specified	EPDS (minor: 9–12; major: > 13)	Prevalence	Minor: 16.7%; major: 15.4%	Medium
Cardillo et al. 2016^85^	Brazil	Adolescents attending basic health units in an urban area of São Paulo	13–19^b^	72	Within 4 months	EPDS (> 13)	Prevalence	20.80%	High
Samankasikorn et al. 2016^a^^35^	USA	Pregnant adolescents recruited from three locations (two urban, one rural)	Teenagers^b^	66	3 months	EPDS	Mean (SD)	4.48 (3.95)	Medium
Surkan et al. 2016^86^	Bangladesh	Married women aged 13–44 years from two rural districts	13–19	21 294	6 months	Modified from the PHQ and the CES-D	Prevalence	1–2 symptoms: 36.2%; 3–5 symptoms: 11.8%	Medium
Anderson & Strickland 2017^87^	USA	Hispanic adolescents attending two postnatal care units at large, public hospital	13–19^b^	66	Within 72 hours	EPDS (> 10)	Prevalence	16.70%	Medium
Eastwood et al. 2017^88^	Australia	Mothers of all infants born in public health facilities within the South Western Sydney Local Health District and the Sydney Local Health District in 2014	14–19	404	Within 6 weeks	EPDS (> 13)	Prevalence	2.50%	High
Faisal-Cury et al. 2017^a^^37^	Brazil	Pregnant women recruited from 10 public primary care clinics in São Paulo	16–19	147	11 months	SRQ-20 (> 7)	Prevalence	15%	Medium
Islam et al. 2017^89^	Bangladesh	Attendees of vaccination centres within two sub-districts of Chandpur district	14–18	106	Within 6 months	EPDS (> 10)	Prevalence	26.00%	Medium
Kim et al. 2017^90^	Canada	Nationwide survey	15–19	23 945	Within 1 year	CES-D (higher score indicates greater presence of symptoms)	Mean (SD)	5.11 (0.43)	High
Mukherjee et al. 2017^a^^91^	USA	Women 2–4 months after delivery, nationwide survey	< 19	< 17 years: 1 724; 18–19 years: 5 229	1 year	PRAMS, Paranoid personality disorder item (> 10)	Prevalence (95% CI)	< 17 years: 14.2 (11.4–17.1)% 18–19 years: 14.8 (13.1–16.6)%	High
Roberts & Hansen 2017^a^^92^	USA	Women enrolled in the military health insurance programme during October 2012–September 2014	12–19	2 212	Within 1 year	Military Health System Management Analysis and Reporting Tool (using ICD-9 codes)	Kaplan–Meier prevalence estimate (95% CI)	8.8 (7.4–10.2)%	Medium
Souza et al. 2017^a^^93^	Brazil	Women with children aged ≤ 3 months attending health-care centres in the Federal District	14–19	958	Within 3 months	EPDS-6 (> 6)	Prevalence (95% CI)	43.3 (40.1–46.5)%	Medium
Anderson & Connolly 2018^94^	USA	Adolescents recruited from two large postpartum care units	13–19^b^	303	72 hours; 3 months; 6–9 months	EPDS (minor: 10–12; major: ≥ 13)	Prevalence	72 hours postpartum: minor, 11.4%; major, 13.5%; 3 months: minor, 15.9%; major, 8.7%; 6–9 months: minor, 7.0%; major, 13.0%	Medium
Salehi-Pourmehr et al. 2018^a^^44^	Iran (Islamic Republic of)	Pregnant women of healthy weight and with BMI ≥ 35	15–19	64	6–8 weeks	EPDS	Median (min, max)	7 (0, 21)	Low
Surkan et al. 2018^a^^45^	Bangladesh	Married women aged 13–44 years from two rural districts	< 19	11 522	Within 6 months	Adapted from PHQ-9 and CES-D	Prevalence	10.40%	Medium
**Anxiety disorder**									
Amr & Hussein Balaha 2010^a^^61^	Saudi Arabia	Primigravid adolescents attending postnatal care within 2 months of delivery	15–19^b^	190	Within 2 months	MINI 5.0	Prevalence	Anxiety disorders: 15.3%; generalized anxiety disorder: 2.6%; social phobia: 3.2%; panic disorder: 2.6%; obsessive–compulsive disorder: 1.1%; post-traumatic stress disorder: 1.1%; agoraphobia: 1.1%	High
East et al. 2012^a^^18^	USA	First-time pregnant adolescents, unmarried, Mexican–American	15–19^b^	100	6 months, 1 year	RCMAS	Mean (SD)	6 months: 2.74 (0.99); 1 year: 2.90 (1.29)	Medium
Salehi-Pourmehr et al. 2018^a^^44^	Iran (Islamic Republic of)	Pregnant women of healthy weight and with BMI ≥ 35	15–19	64	6–8 weeks	BAI II	Median (min, max)	3 (0, 20)	Low
**General mental disorder/ psychological distress**									
Amr & Hussein Balaha 2010^a^^61^	Saudi Arabia	Primigravid adolescents attending postnatal care within 2 months of delivery	15–19^b^	190	Within 2 months	MINI 5.0	Prevalence	22.6%	High
Clarke et al. 2014^95^	Nepal	Women who gave birth during April 2008–April 2011 in a rural community	< 20	1 810	≤ 8 weeks	GHQ (≥ 6)	Prevalence	10.00%	High
**Suicidality**									
Tavares et al. 2012^a^^96^	Brazil	Women giving birth in urban maternity wards in Pelotas during July 2007–March 2008	13–19	181	30–90 days	MINI	Prevalence	13.80%	Medium

BAI: Beck Anxiety Inventory; BDI: Beck Depression Inventory; BMI: body mass index; CDRS-R: Children’s Depression Rating Scale; CES-D: Center for Epidemiologic Studies Depression scale; CI: confidence interval; DSM-IV: Diagnostic and Statistical Manual of Mental Disorders, fourth edition; DSSI/SAD: Delusions-Symptoms-States Inventory: State of Anxiety and Depression; EPDS: Edinburgh Postnatal Depression Scale; GHQ: General Health Questionnaire; ICD: International Statistical Classification of Diseases and Related Health Problems; Kid-SCID: Structured Clinical Interview for DSM-IV Childhood Disorders; KSADS-PL: Schedule for Affective Disorders and Schizophrenia for School-Age Children, present and lifetime version; MINI: Mini-International Neuropsychiatric Interview; PHQ-2/-9: 2-/9-item Patient Health Questionnaire; PRAMS: Pregnancy Risk Assessment Monitoring System; PREP: Parenting Responsibility and Emotional Preparedness; RCMAS: Revised Children’s Manifest Anxiety Scale; SD: standard deviation; SRQ-20: 20-item Self-Reporting Questionnaire.

^a^ This publication is also included in other table(s).

^b^ Study population was adolescents only.

^c^ Sample included males.

^d^ Weighted sample.

^e^ Our calculations.

**Table 4:** Systematic review of adolescent mental health following sexual and reproductive health events, 2020; studies of both pregnancy and postpartum period

Author, year	Country	Study population	Age (years)	No. adolescents	Sexual and reproductive health event time frame	Assessment tool	Outcome reported	Outcome estimate	Quality rating
**Depression**									
Connelly et al. 2013^97^	USA	Women receiving routine maternal health services any time during the perinatal period (including the 6-week postpartum visit) at 10 obstetric/gynaecologic clinics in San Diego	< 18, 18–19	262 (< 18 years: 87; 8–19 years: 175)	Antenatal, 6 weeks postpartum	EPDS (≥ 10)	Prevalence	< 18 years: 16.1% (14/87); 18–19 years: 20.6% (36/175)^a^	High
**Suicidality**									
Palladino et al. 2011^98^	USA	Nationwide, female victims of pregnancy-associated violent deaths of reproductive age during 2003–2007	15–19	456 478	Pregnancy, 1 year postpartum	National death records; cause of death	Prevalence	0.0026%	Low

EPDS: Edinburgh Postnatal Depression Scale.

^a^ Our calculations.

Only three studies focusing on the postpartum period alone reported on anxiety (as well as depression; Table 3), one of which reported the prevalence of any anxiety disorder as 15.3%.^61^ The other two studies provided results in the form of mean or median scores.^18^^,^^44^ Two studies reported on psychiatric disorders or psychological distress during the postpartum period (one of which also reported on depression), reporting a prevalence of 22.6%^61^ and 10.0%,^95^ respectively. Finally, one study reported the prevalence of suicidal risk during this period as 13.8%.^96^

Of the 13 studies that determined the risk of depression during the postpartum period in adolescents compared with other age groups, nine studies identified an increased risk of depression for adolescents. A study reporting on postpartum anxiety (as well as depression) did not find adolescents to be at a higher risk than other age groups (Table 5).^101^ However, a study examining suicide risk during this period found adolescents to be at the greatest risk of suicide compared with other age groups.^96^

**Table 5:** Systematic review of adolescent mental health following sexual and reproductive health events, 2020; studies comparing adolescents with other age groups during postpartum period

Author, year	Country	Assessment tool	Sexual and reproductive health event time frame	Outcome reported	Adolescent age (years)	No. adolescents	Adolescent outcome estimate	No. in comparison group	Comparison group outcome estimate(s)	Quality rating
**Depression**										
Surkan et al. 2018^a^^45^	Bangladesh	Adapted from PHQ-9 and CES-D	6 months	Adjusted RR (95% CI)	< 20	12 862	1.0 (–)	18 543	20–29 years: 1.09 (1.02–1.20); ≥ 30 years: 1.44 (1.29–1.61)	Medium
Almeida et al. 2012^a,b^^9^	Brazil	EPDS (≥ 13)	Within 1 year	Crude PR (95% CI)	13–19	63	1.27 (0.61–2.64)	395	20–34 years: 1.0 (–); ≥ 35 years: 1.69 (0.85–3.38)	Medium
Chittleborough et al. 2012^a^^71^	United Kingdom	EPDS (> 12)	8 weeks	Crude OR (95% CI)	< 20	655	1.83 (1.38–2.41)	9415	> 20 years: 1.0 (–)	Medium
Kingston et al. 2012^99^	Canada	EPDS (≥ 13)	Within 3 months	Adjusted OR (95% CI)	15–19	2 262	2.29 (1.48–3.54)	73 797	20–24 years: 1.43 (1.03–1.99); ≥ 25 years: 1.0 (–)	Medium
Silva et al. 2012^a^^72^	Brazil	EPDS (≥ 13)	30–60 days	Crude PR (95% CI)	13–19	215	1.07 (0.74–1.57)	804	20–24 years: 0.88 (0.61–1.28); 25–29 years: 0.70 (0.47–1.06); 30–45 years: 1.0 (–)	Medium
de Castro et al. 2015^a^^79^	Mexico	EPDS (≥ 12)	Within 9 months	Adjusted OR (95% CI)	14–19	120	1.3 (0.5–2.9)	484	≥ 20 years: 1.0 (–)	High
Kingsbury et al. 2015^a^^80^	Australia	DSSI/SAD-7	6 months	Adjusted OR (95% CI)	14–19	345	1.73 (1.20–2.50)	2646	20–29 years: 1.38 (1.06–1.80); ≥ 30 years: 1.0 (–)	Medium
Suh et al. 2016^100^	USA	PRAMS	Within 9 months	Adjusted OR (95% CI)	< 18	290	1.0 (–)	5259	Mild paranoid personality disorder 19–24 years: 0.95 (0.65–1.37); 25–34 years: 0.93 (0.62–1.41); ≥ 35 years: 0.87 (0.54–1.41) Severe paranoid personality disorder 19–24 years: 1.11 (0.59–2.08); 25–34 years: 1.05 (0.52–2.15); ≥ 35 years: 0.84 (0.36–1.95)	Medium
Mukherjee et al. 2017^a^^91^	USA	PRAMS	Within 1 year	Adjusted OR (95% CI)	< 17, 18–19	< 17 years: 1724; 18–19 years: 5229	< 17 years: 0.91 (0.89–0.94); 18–19 years: 0.93 (0.92–0.94)	84 300	20–24 years: 0.99 (0.98–1.00); 25–29 years: 1.0 (–); 30–34 years: 0.97 (0.97–0.98); 35–39 years: 0.89 (0.88–0.90); ≥ 40 years: 1.07 (1.05–1.09)	High
Roberts & Hansen 2017^a^^92^	USA	Military health system management analysis and reporting tool (using ICD-9 codes)	Within 1 year	Kaplan–Meier prevalence estimate (95% CI), adjusted HR (95% CI)	12–19	2 212	Kaplan–Meier prevalence: 8.8 (7.4–10.2)%; Adjusted HR: 2.03 (1.50–2.76)	73 316	Kaplan–Meier prevalence 20–24 years: 6.8 (6.2–7.4)%; 25–29 years: 5.1 (4.7–5.5)%; 30–34 years: 3.9 (3.7–4.1)%; 35–39 years: 3.9 (3.5–4.3)%; ≥ 40 years: 3.9 (2.9–4.9)% Adjusted HR 20–24 years: 1.33 (1.02–1.74); 25–29 years: 1.15 (0.88–1.49); 30–34 years: 0.94 (0.72–1.23); 35–39 years: 0.99 (0.75–1.31); ≥ 40 years: 1.0 (–)	Medium
Signal et al. 2017^a^^101^	New Zealand	EPDS (≥ 13)	4–6 weeks; 12 weeks	Crude OR (95% CI)	16–19	65	4.80 (1.61–14.27)	1079	20–24 years: 3.66 (1.38–9.71); 25–29 years: 2.18 (0.85–5.61); 30–34 years:1.16 (0.46–2.94); 35–39 years: 1.54 (0.60–3.94); 40–46 years: 1.0 (–)	Medium
Silverman et al. 2017^102^	Sweden	ICD-10 codes	Within 1 year	Adjusted RR (95% CI)	15–19	17 823	1.48 (1.26–2.72)	689 878	20–24 years: 1.12 (1.02–1.22); 25–29 years: 1.0 (–); 30–34 years: 1.11 (1.03–1.20); 35–39 years: 1.25 (1.13–1.37); ≥ 40 years: 1.25 (1.07–1.47)	Medium
Souza et al. 2017^a^^93^	Brazil	EPDS-6 (≥ 6)	Within 3 months	Adjusted OR (95% CI)	14–19	958	3.02 (2.49–3.66)	9510	> 20 years: 1.0 (–)	Medium
**Anxiety**										
Signal et al. 2017^a^^101^	New Zealand	EPDS (≥ 6)	4–6 weeks; 12 weeks	Crude OR (95% CI)	16–19	65	1.98 (0.78–5.05)	1079	20–24 years: 2.39 (1.07–5.36); 25–29 years: 0.94 (0.45–1.99); 30–34 years: 0.94 (0.45–1.99); 35–39 years: 0.89 (0.41–1.94); 40–46 years: 1.0 (–)	Medium
**Suicidality**										
Tavares et al. 2012^a^^96^	Brazil	MINI	30–90 days	Crude PR (95% CI)	13–19	181	1.92 (0.80– 4.63)	724	20–34 years: 1.54 (0.69–3.46); 35–45 years: 1.0 (–)	Medium

CES-D: Center for Epidemiologic Studies Depression scale; CI: confidence interval; DSSI/SAD: Delusions-Symptoms-States Inventory: State of Anxiety and Depression; EPDS: Edinburgh Postnatal Depression Scale; HR: hazard ratio; ICD: International Statistical Classification of Diseases and Related Health Problems; MINI: Mini-International Neuropsychiatric Interview; OR: odds ratio; PHQ-9: 9-item Patient Health Questionnaire; PR: prevalence ratio; PRAMS: Pregnancy Risk Assessment Monitoring System; RR: relative risk.

^a^ This publication is also included in other table(s).

^b^ Sample included males.

### Abortion

We list the three included studies on mental health outcomes following an induced abortion among adolescents in Table 6. Two studies reported that the abortion took place within 12 weeks gestation;^103^^,^^104^ the third study did not specify when the abortion took place.^67^ Two studies reported the prevalence of depressive symptoms as 16.1%^67^ and 85.0%,^103^ reporting at least mild symptoms of depression. One study reported an average depression score^104^ and another provided mean anxiety scores.^103^

**Table 6:** Systematic review of adolescent mental health following sexual and reproductive health events, 2020; studies of abortion

Author, year	Country	Study population	Age (years)	No. adolescents	Sexual and reproductive health event timeframe	Assessment tool	Outcome reported	Outcome estimate	Quality rating
**Depression**									
Warren et al. 2010^a^^67^	USA	Nationally representative survey of United States adolescents in secondary school in 1994–1995	12–17^b^	69	Post-abortion; 1 year later	CES-D (> 22)	Prevalence	Post-abortion: 16.1%; 1 year later: 14.1%	High
Zulčić-Nakić et al. 2012^103^	Bosnia and Herzegovina	Adolescents without history of psychiatric disease who had an abortion up to 12th week of pregnancy at a university hospital	14–19^b^	120 (60 with abortion)	Abortion	BDI (mild: 11–16; borderline: 17–20; moderate: 21–30; serious: 31–40; extremely: ≥ 41)	Prevalence	Mild: 6.7%; borderline: 3.3%; moderate: 40.0%; serious: 16.7%; extremely: 18.3%	High
Pereira et al. 2017^104^	Portugal	Women who had an abortion on request up to 12th week of pregnancy at one of 16 centres	14–19	177	Abortion	EPDS (> 9)	Mean (SD)	11.27 (5.76)	Medium
**Anxiety**									
Zulčić-Nakić et al. 2012^103^	Bosnia and Herzegovina	Adolescents without history of psychiatric disease who had an abortion up to 12th week of pregnancy at a university hospital	14–19^b^	120 (60 with abortion)	Abortion	STAI (higher scores indicate greater anxiety)	Mean (SD)	STAI-T: 59.8 (8.9) STAI-S: 57.9 (9.7)	High

BDI: Beck Depression Inventory; CES-D: Center for Epidemiologic Studies Depression scale; EPDS: Edinburgh Postnatal Depression Scale; SD: standard deviation; STAI: State–Trait Anxiety Inventory; STAI-S: State–Trait Anxiety Inventory - State; STAI-T: State–Trait Anxiety Inventory - Trait.

^a^ This publication is also included in other table(s).

^b^ Study population was adolescents only.

We did not identify any studies that compare mental health outcomes among adolescents with those of other age groups post-abortion.

## Discussion

Our systematic review of the literature reporting on the mental health outcomes among adolescents after key sexual and reproductive health events reveals a very high prevalence of mental ill-health. This finding is particularly true for depression, the most commonly assessed mental health outcome in our review. The prevalence of depression varies widely between cultures;^105^ however, the WHO World Health Survey of 60 countries found an average annual prevalence of 3.2% in participants without comorbid physical disease.^106^ Global estimates indicate that 10% of pregnant women and 13% of postpartum women (of all ages) experience a mental disorder.^107^ However, among studies reporting the prevalence of depression in this review, 92.9% (26/28) of studies of pregnant adolescents, and 73.0% (27/37) of studies of postpartum adolescents, reported a higher figure than these global estimates.

Our results indicate a high prevalence of depression during pregnancy across all age groups, highlighting the importance of recognizing the mental health needs of all women during pregnancy. By contrast, while not conclusive, comparison data for the postpartum period suggests that there may be a higher prevalence of depression among postpartum adolescents than among postpartum women of older age groups. This higher prevalence among adolescents may be the effect of the challenges facing adolescent mothers in caring for an infant, such as financial burden, social isolation from peers, limited support and the detrimental consequences of being excluded from further education. Our findings also corroborate what is known about the adverse effects of early marriage and the often-ensuing expectation to give birth.^108^

Regardless of the nature of the sexual and reproductive health event, it appears that suicidality may be a concern for adolescents. Although only two studies provided comparison data on suicidality between age groups, both showed the risk to be significantly higher among adolescents compared with older age groups.^56^^,^^96^ Such findings are consistent with other research suggesting that adolescent mothers may have an elevated risk of suicidal ideation.^109^ This result emphasizes the need for both further research and enhanced services.

Although the assessments of mental health conditions other than depression were relatively limited, we found a high burden of anxiety and common mental health disorders among adolescents following sexual and reproductive health events. We were surprised to discover only a few studies reported on anxiety,^110^ given that depression and anxiety are often comorbid and that the prevalence of anxiety is high among adolescents; a study based in the USA found that 31.9% of adolescents have an anxiety disorder.^111^ Our findings point to the need for research on the full spectrum of mental disorders to fully understand the mental ill-health burden experienced by adolescents following such health events, concurring with other calls for research on a broader range of mental health conditions among perinatal women of all ages.^112^^,^^113^

Our findings reveal another gap in the mental health literature; we found only three articles that reported on abortion and zero articles reporting on the prevalence of sexually transmitted infections among adolescents. Our search did identify 46 articles reporting on HIV; however, we had to exclude these from our review because HIV was either vertically transmitted (i.e. not the result of an unprotected sexual event) or the reviewers were unable to distinguish between mental health outcomes for vertically and horizontally transmitted HIV. Furthermore, while our review included studies on both males and females, nearly all of the included studies (with the exception of two) focused on adolescent females. While this finding is understandable for the reproductive events of pregnancy and abortion, there is an obvious need for more research on the mental health of adolescent males as a result of relevant sexual and reproductive health events (e.g. sexually transmitted infection/HIV, new fatherhood).

We also found few studies describing mental health outcomes following an abortion, highlighting another important area for further research. A study^67^ found depression rates to be lower among female adolescents with unintended pregnancies who had an abortion, compared with those who delivered, recorded either one year after abortion or delivery. Although this is only one study, this result supports the notion that when afforded the right to choose, women who elect to have an abortion rarely regret it.^114^

While our systematic review has several strengths – such as considering literature published over an entire decade, the thoroughness of the search, and the double-checking of data extraction and quality scoring results – there are some limitations. We did not examine risk factors for mental health outcomes among adolescents: there may be certain demographic factors (e.g. age, income, ethnicity, education level) within the adolescent population that could increase (i) their vulnerability to mental health challenges; (ii) their potential to experience a sexual and reproductive health event; and/or (iii) the incidence and severity of any resulting mental health outcomes. While we were interested in mental health outcomes following a sexual and reproductive health event, we could not always be certain about the exact temporal relationship; it is possible that mental health issues may have increased vulnerability to the particular sexual and reproductive health event. We also excluded qualitative studies from this review; although qualitative data can provide a rich understanding of the impact of such health events on the mental health and well-being of adolescents, a mixed-methods systematic review was beyond our scope. Finally, our quality assessment tool did not undergo a formal psychometric evaluation; however, we based our quality assessment tool on an existing and widely used instrument, which was deemed to have content validity and was used by two authors independently. We felt that this instrument was adequate for our objective of providing an explicit indication of study quality, rather than a precise measurement.

We identified methodological issues in many of the included studies. Most studies used assessment tools that screen for the severity of symptoms, but cannot provide a mental health diagnosis. We observed that a broad range of assessment tools were used, as well as different cut-off points for the same tool between different studies. For example, one study^31^ used a Center for Epidemiologic Studies Depression scale (CES-D) cut-off of ≥ 16, whereas another study^16^ used one of ≥ 24. Almost half of the included studies used the Edinburgh Postnatal Depression Scale (EPDS); of these studies, many (21/40) used a cut-off of ≥ 13 although others used scores of 10^15^ or 9.^27^ The field of mental health would benefit from the streamlining of screening tools and cut-offs used, which would also encourage research that uses diagnostic tools to confirm mental health conditions rather than solely identifying symptom severity. The use of rigorous clinical diagnostic interviews to assess mental health disorders would provide a clearer clinical picture of the mental health burden among adolescents who have experienced a sexual and reproductive health event.

To address the mental health burden associated with pregnancy or sexually transmitted infections, future work should identify effective psychosocial interventions that can be made available to adolescents who experience such a health event. There is evidence that adolescents often do not use mental health services, so these efforts should consider how to successfully connect identified adolescents with the care they need.^115^^,^^116^ Additional research could also identify risk and protective factors in adolescents who have experienced such a health event by comparing those who developed mental health issues with those who did not; this strategy may help to determine whether targeted interventions can build resiliency among adolescents who experience such a health event. Similarly, research is also needed to explore the extent to which adolescents experience adverse mental health outcomes, comparing those who previously experienced a sexual and reproductive health event with those who did not. As a promising step, WHO published the *Guidelines on mental health promotive and preventive interventions for adolescents* in 2020, with recommendations targeting all adolescents and particularly vulnerable groups.^117^

In conclusion, considering the mental health burden that adolescents are experiencing, we now need to develop, implement and evaluate appropriate services to support the adolescent population. Similarly, sexual and reproductive health services and information should be accessible to adolescents to address their needs and help to prevent any unintended outcomes that could have consequences for their mental health. Health-care providers encountering adolescents following such a health event must be prepared to screen for, and address, any mental health concerns. Going forwards, we recommend that mental health care is considered an integral part of sexual and reproductive health service provision.
